# Managers’ perceptions of organizational readiness for change within disability healthcare: a Swedish national study with an embedded mixed-methods approach

**DOI:** 10.1186/s12913-025-12808-4

**Published:** 2025-05-06

**Authors:** Anette Granberg, Lars-Olov Lundqvist, Anna Duberg, Marie Matérne

**Affiliations:** 1 University Health Care Research Centre, Faculty of Medicine and Health, Orebro University, S-huset, vån 2, Orebro, SE-70185 Sweden; 2 School of Behavioural, Social and Legal sciences, Orebro University, Orebro, Sweden

**Keywords:** Managers, Organizational readiness, Implementation, Disability, Mixed-methods approach

## Abstract

**Background:**

People with disabilities experience significant health inequities compared with the general population. Addressing these inequities requires the development and implementation of tailored interventions, but a gap often exists between recommended best practices and the actual care provided. Successful implementation is complex, involving multiple organizational factors. Assessing organizational readiness for change is crucial to overcome barriers and improve health outcomes for people with disabilities. This study aims to examine managers’ perceptions of their organization’s readiness for change regarding the implementation of interventions within disability healthcare in Sweden.

**Methods:**

This descriptive cross-sectional study employs an embedded mixed-methods approach. The primary approach for the overall study is based on quantitative data, while qualitative data is analyzed to provide supplementary deepened information. Both types of data were collected simultaneously through a web-based survey. The data analysis involves various statistical techniques for the quantitative data and inductive content analysis for the qualitative data.

**Results:**

Several key factors influence managers’ perceptions of their organization’s readiness for change, including *gender*, *age*, *tenure*, *organizational type*, *managerial level*, and *experience.* Enabling factors for implementation include *trust-based leadership*, *staff involvement*, *motivation*, and *engagement*. Barriers include *complex processes*, *lack of support*, *resistance and fear*, and *insufficient time and resources.*

**Conclusions:**

This study highlights the complexity of organizational readiness for disability healthcare interventions, shaped by both individual and organizational factors. In particular, managerial characteristics, organizational dynamics, and resource availability play key roles. These findings suggest that a comprehensive strategy can strengthen healthcare organizations’ ability to navigate implementation challenges effectively.

**Supplementary Information:**

The online version contains supplementary material available at 10.1186/s12913-025-12808-4.

## Contributions to the literature

- This research advances the theory development of implementation in the field of disability services and contributes to a broader understanding of organizational readiness for change regarding the implementation of interventions in the context of disability services.
- The results help identify areas where additional support or resources may be needed to facilitate successful implementation; they also guide the development of targeted strategies in order to enhance organizational readiness for change within the disability context.
- The findings can be used to inform policy decisions and resource allocation for implementation in Swedish disability healthcare.

## Background

People with disabilities have unequal access to healthcare, greater unmet healthcare needs, and poorer levels of health compared with the general population [1]. These disparities stem from various barriers, including physical inaccessibility, communication challenges, and attitudinal biases among healthcare providers [2]. Sweden has introduced several innovative approaches to reduce address healthcare inequities for people with disabilities. One key initiative is the use of personal assistance programs, which provide individualized support for daily activities [3]. However, research highlights the need for better coordination between healthcare and social services, particularly for individuals with intellectual and developmental disabilities (IDD) [4]. Challenges include insufficient adjustments to meet individual needs, and fragmented systems [4]. For instance, Degerstedt et al. [5] found disparities in care for children with Cerebral Palsy in Sweden, with interventions unevenly distributed across different geographical regions. Despite these efforts, ensuring consistent implementation of disability healthcare policies across the country, particularly in rural areas [6], remains a challenge.

To address these inequities, organizations must develop and implement interventions that are both effective and tailored to the unique needs of individuals with disabilities [7]. However, there is an “implementation gap” between recommended best practices and the care that is actually provided [8], resulting in a risk that people with disabilities may not receive the care they need [1]. To bridge this implementation gap, there is an increased demand for shared responsibility and collaboration between regional and local healthcare organizations, health care providers, and health and social care system to meet the overall needs of this patient group [9]. When successfully implemented, interventions can lead to improved health outcomes, increased patient satisfaction, and more efficient use of healthcare resources, ultimately enhancing the quality of care for people with disabilities [10].

A problem faced by healthcare organizations is that the implementation of interventions can be complex, involving collaboration among different professions, teams, managers, patients, relatives, and nursing staff [11]. The perception of workplace culture, team cooperation, leadership effectiveness, and existing organizational conditions can determine whether and how the implementation of an intervention occurs [12]. These factors operate at multiple levels within an organization and ultimately influence whether an implementation succeeds or fails [13]. The implementation of innovations for people with disabilities is specifically influenced by various factors such as organizational factors (e.g., management support, resources), technology-related factors (e.g., adaptability, complexity) [14], human factors (e.g., staff attitudes, skills), collaborative factors (e.g., relationships between professionals and individuals) and contextual factors (e.g., policies, societal context) [1]. These factors interact and can impact the implementation process across different phases of implementation including preparation, adoption, implementation, and sustainment, each phase is influence by specific combinations of factors [15].

Given the complexity of these interacting factors, the concept of organizational readiness for change (ORC) has emerged as a critical consideration in implementation efforts. It has been argued that up to 50% of unsuccessful implementations fail due to a lack of ORC [16]. In their ORC theory, Weiner et al. [16] posit that readiness is determined by the extent to which individuals feel committed to change, are confident in the collective ability to change, and value the change as important and worthwhile, along with there being sufficient resources for change in the organization. This underscores the importance of understanding and assessing organizational readiness before initiating the implementation of an intervention [17]. However, organizational readiness is influenced by several factors, including the characteristics of the intervention or new practice being implemented, the process of implementation, involving the strategies and methods used to introduce and integrate the change [18]. Furthermore, the context in which the change occurs relating to environment, culture, past experiences with change, and the characteristics of the individuals involved such as skills, attitudes, beliefs and commitment of employees’ ability to implement the change, making it challenging to study [18].

Nevertheless, previous research provides some clues. For instance, leadership with consistent messaging and actions—such as sharing knowledge, providing opportunities, or providing social interactions among staff—has been shown to promote a sense of high readiness for implementation [17, 19]. Common values and experiences, such as recruitment and staff turnover, have also been shown to play a role in readiness. High turnover rates can hinder readiness by increasing training demands, causing a loss of organizational knowledge, and reducing the fidelity of evidence-based practices. However, turnover can also have a positive impact by introducing new perspectives and skills that enhance readiness [20]. Factors such as age, education, and gender can also influence implementation readiness [21]. Older employees may bring valuable experience, while younger employees’ might be more adaptable to new technologies [22]. Additionally, higher levels of education can improve employees’ ability to understand and implement new practices. Some studies [23, 24] suggest that gender may be linked to factors like readiness for change and job involvement, though the specific impact varies depending on the organizational context. These factors are particularly relevant in healthcare. However, the readiness of organizations to implement interventions in disability healthcare remains underexplored [25].

In Sweden, disability healthcare faces significant challenges that may affect readiness for implementation. The country’s decentralized and fragmented healthcare system requires extensive coordination, yet often struggles to provide safe, equitable healthcare for individuals with disabilities. This lack of coordination can lead to disparities in access to care [4]. Therefore, organizational readiness is essential for improving coordination between healthcare and social services [26]. It can create better opportunities for coordination and help address health inequalities. People with disability frequently encounter structural barriers to timely and adequate healthcare. By improving organizational readiness, healthcare providers can help develop and implement strategies to overcome these barriers. To gain a better understanding of the enabling and/or hindering factors in the implementation of interventions, it is necessary to measure the readiness for change in organizations [27].

Since, managers play a vital role in leading and facilitating change within their teams, their perceptions of readiness can directly impact decisions regarding time, personnel, and financial resources allocated to implementing interventions [28]. By examining how prepared managers are before acting and how they respond to implementation, we can gain insight into their perceived level of readiness. This focus on readiness makes it possible to understand the potential challenges and opportunities in implementing interventions within this context. Therefore, this study aimed to examine managers’ perceptions of their organizations’ readiness for change regarding the implementation of interventions within disability healthcare in Sweden.

## Methods

### Study design

To provide both a broad quantitative overview of organizational readiness and qualitative in-depth insights into enabling and barrier factors affecting the implementation of interventions, this descriptive cross-sectional study employs an embedded mixed-methods approach [29, 30]. Quantitative and qualitative data were collected simultaneously through a web-based survey and then analyzed separately. The primary approach for the overall study was based on quantitative data, while qualitative data was analyzed to provide supplementary information. The STROBE checklist [31] for cross-sectional studies (Additional file 1) and the Good Reporting of a Mixed-Methods Study (GRAMMS) checklist [32] were used to guide and report this study (Additional file 2).

### Study setting

In Sweden, disability services are provided and managed by 21 regions and 290 municipalities. The regions are responsible for healthcare services such as habilitation services, whereas the municipalities are responsible for support and care to facilitate the everyday lives of people with disabilities, such as personal assistance and residential care services. In municipalities, private companies can provide these services. In 2023, 79 100 people with disabilities in Sweden received service under the Act concerning Support and Service for Persons with Certain Functional Impairments (LSS) [33]. This number has increased by 27% since 2010. The target population for disability care in Sweden includes both individuals residing in long-term care facilities and those living independently who require support [34].

### Study participants

A purposive sampling was used to select participants based on predetermined criteria that aligned with the study aims, focusing on a specific characteristic of the target population. To be included in the study, participants had to be managers at various levels (area managers, operational managers, and unit managers or similar) responsible for implementing interventions for people over 18 years of age with disabilities. These managers worked within regional habilitation services, municipalities, and private organizations in Sweden including those providing personal assistance and residential care. In addition, the manager was required to have been employed at the current workplace for at least three months.

### Data collection

**Fig. 1:**
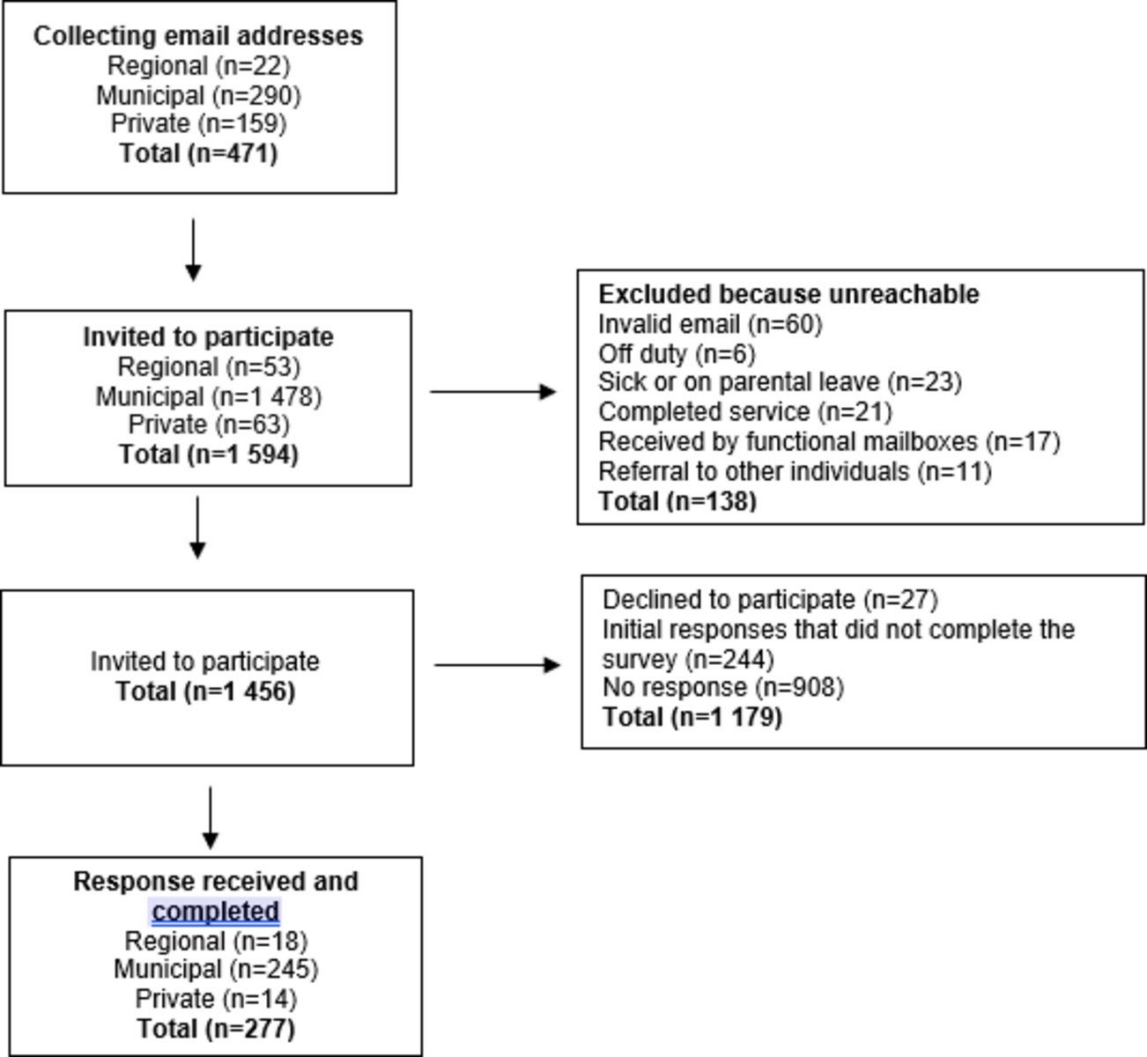
Flowchart of the response rate of the study

During November and December 2023, email addresses of managers across Sweden were collected (*n* = 471). Within regional and private organizations, we obtained addresses by visiting the organizations’ websites; within municipalities, we approached through their general function mailboxes. We then sent an email to the collected addresses, asking the managers to select and provide the email addresses of relevant respondents in their organizations. By the end of January 2024, the managers had selected and provided us with 1 594 email addresses (Fig. 1). An invitation to respond to the survey was then sent out to all the obtained addresses (*n* = 1 594) in January 2024 via an email containing a link to a web-based survey, along with information about the study purpose and consent to participate. We also compiled an appendix that described the research project in more detail. The information emphasized that participation was voluntary and could be canceled at any time by the respondent. The survey was anonymous, and the choice of quotes was made carefully so that no respondent could be identified. A reminder to answer the survey was sent three times, 14 days apart. The survey was closed on March 31, 2024.

### Measures

The Texas Christian University Organizational Readiness for Change (TCU-ORC) survey [35], developed by Lehman et al. [36] was chosen to measure organizational readiness for change. The TCU-ORC survey is a suitable tool for assessing ORC in various healthcare contexts, including disability care. Its ability to identify functional barriers to organizational change can be valuable in implementing new interventions in disability healthcare settings [36]. A pre-test of the survey was conducted on four people within regional, municipal, and private organizations to obtain feedback on the overall content and their experiences of the survey. Some adjustments were made to the survey’s introduction to enhance response rates and data quality without modifying the core questionnaire. These changes aimed to engage respondents and set clear expectations for the survey.

#### Socio-demographic and organizational characteristics

The questionnaire measured socio-demographic variables such as age, gender, education level, and primary profession, in addition to being a manager. The managers were asked to provide the approximate number of clients served and the number of employees for which they were responsible. They answered questions about their years of experience and years at their current workplace on a 5-point scale (0–1 year, 2–5 years, 6–9 years, 10–19 years, or more than 20 years). Managerial roles were measured by identifying the role (area manager, operational manager, head of unit, or other) and were recoded to a two-category variable (managers directly overseeing staff or managers of managers). Type of organization (regional, municipal, or private) and service provided (habilitation, personal assistant, or residential care) were measured using three-category variables. Finally, the primary service area was measured as a four-category variable based on population size (urban area > 200 000 inhabitants, suburban area > 40 000 inhabitants, suburban area > 15 000 inhabitants, or rural area < 15 000 inhabitants) (Table 1).

**Table 1:** Socio-demographic and organization characteristics

Variables	Frequency (%)	Mean score (range; SD)
Age		49.8 years (25–73; 9.8)
Gender		
Women	228 (82.3)	
Men	49 (17.6)	
Level of education		
Compulsory or high school	9 (3.2)	
College/university	261 (94.2)	
Other	7 (2.5)	
Core profession		
Care or nursing assistant	13 (4.7)	
Social worker/counselor	86 (31)	
Occupational therapist	10 (3.6)	
Physiotherapist	8 (2.9)	
Nurse	17 (6.1)	
Other	143 (51.6)	
Years of managerial experience		
0–1 year	9 (3.2)	
2–5 years	43 (15.5)	
6–9 years	36 (13.0)	
10–19 years	84 (30.3)	
More than 20 years	105 (37.9)	
Current workplace		
0–1 year	49 (17.7)	
2–5 years	107 (38.6)	
6–9 years	51 (18.4)	
10–19 years	41 (14.8)	
More than 20 years	29 (10.5)	
Number of clients		292 (0–14 000; 1356.8)
Positions		
Managers of managers	49 (17.7)	
Managers directly overseeing staff	240 (86.6)	
Number of employees		54 (0–1500; 124.9)
Type of organization		
Regional level	18 (6.5)	
Municipality level	245 (88.4)	
Private level	14 (5.1)	
Services		
Habilitation	41 (12)	
Personal assistance	82 (24)	
Residential care	215 (64)	
Primary service area		
Urban area, > 200 000 inhabitants	48 (17.3)	
Suburban area, > 40 000 inhabitants	88 (31.8)	
Suburban area, > 15 000 inhabitants	81 (29.2)	
Rural area, < 15 000 inhabitants	60 (21.7)	

#### *The TCU-ORC*

The TCU-ORC survey [35] was used to measure managers’ perceptions of their organization’s readiness for change regarding the implementation of interventions within disability healthcare. The TCU-ORC comprises four key domains: *Motivation for Change*,* Resources*,* Staff Attributes*, and *Organizational Climate*. Each domain contains 20 multiple scales and 115 items to measure organizational readiness. Each item is rated on a 5-point Likert scale, ranging from “Strongly disagree” (1) to “Strongly agree” (5). The scores range from 10 to 50 for each scale, with a midpoint of 30. Scores above 30 indicate stronger levels of agreement, while scores below 30 indicate stronger levels of disagreement. The *Motivation for Change* domain includes the scales *Program Needs*, *Training Needs*, and *Pressure for Change* (i.e., to what degree managers perceive the organization’s need for guidelines and training, and where the pressure to change comes from). The *Resources* domain covers the scales *Offices*,* Staffing*,* Training*,* Equipment*, and *Internet Access* (i.e., physical infrastructure such as facilities, staff patterns, and training, along with IT resources). The *Staff Attributes* domain includes the scales *Growth*,* Efficiency*,* Influence*,* Adaptability*, and *Satisfaction*, providing insights into the workforce’s capacity for change and innovation within the organization. The *Organizational Climate* domain covers the scales *Mission*,* Cohesion*,* Autonomy*,* Communication*,* Stress*,* Change*, and *Leadership*, reflecting the overall organizational mission and goal to promote the identification of needs and facilitate change processes to improve service functioning. In the original validation study by Lehman et al. [36], the TCU-ORC showed good internal consistency reliability across its domains and scales. The Cronbach’s alpha coefficients (α) for the main domains were as follows: Motivation for Change: α = 0.84, Resources: α = 0.76, Staff Attributes: α = 0.72 and Organizational Climate: α = 0.82. The TCU-ORC survey has adequate validity and reliability [37] and has been widely used in multiple studies and settings, especially in the United States, to assess organizational readiness for implementing new practices and interventions [38, 39]. In Sweden, the TCU-ORC has been used in studies on middle managers, operational staff in social care, and personnel at private, municipal, and state institutions [40, 41]. The TCU-ORC questionnaire used in the present study was translated into Swedish in 2009 [42]. The use of the TCU-ORC questionnaire in this study was mandatory.

#### Open-ended questions

Two open-ended questions were included at the end of the questionnaire, with space for answers. These questions focused on potential challenges and opportunities in implementing interventions within this context. The qualitative data served as complementary, in-depth information to the primary quantitative data. By incorporating interview quotes and categories, it provided context and richer insights into managers’ perceptions of their organization’s readiness. The first question addressed managers’ experiences with factors enabling the implementation of interventions, while the second focused on their perceptions of barriers. These questions were not mandatory.

### Data analysis

#### Statistical analysis

Firstly, a bivariate analysis (a one-way analysis of variance [ANOVA] and a correlation analysis for categorical and continuous variables, respectively) was used to examine the statistical relationship between the socio-demographic and organization characteristics (independent variables) and the TCU-ORC responses (dependent variables). Tukey’s range test was conducted to interpret the statistical significance of the difference between the means selected for comparison. Secondly, separate linear regression models that included all variables significant at the bivariate level were developed to examine the relationship between a dependent variable and one or more independent variables. To assess the assumption of normality in the linear regression analysis, residuals were evaluated graphically using quantile–quantile (Q-Q) plots and analysed for skewness and kurtosis. The residuals showed no substantial deviation from the reference line, and skewness and kurtosis remained within the commonly accepted ± 2 range, indicating no significant departure from normality. Multicollinearity was assessed using the variance inflation factor (VIF), with all values remaining below 10, confirming the absence of multicollinearity. All statistics were conducted using SPSS Version 29 [43].

#### Qualitative analysis

We used a content analysis with an inductive approach inspired by Granheim and Lundman [44], with a manifest analysis, and stayed close to the text in order to describe the visible and obvious in the text. The first author (AG) started by coding the open-ended comments in the qualitative data and grouping them into categories. The comments from the respondents were already brief and condensed, so we combined the condensing of meaning units with the coding. AG and MM independently created categories by putting several sub-categories together. The sub-categories and categories were then discussed between the two authors until consensus was reached. The final step involved quantifying how many comments were in each category, in order to enhance and support the structure of the analysis, in line with Krippendorff’s approach to content analysis [45]. This enabled us to identify the most common categories raised by the respondents and detect unusual or unexpected patterns that may require further investigation, potentially leading to new insights or research questions. NVivo software was used for the qualitative analysis [46].

## Results

### Sample characteristics

As shown in Fig. 1, among the 1456 managers who were invited to participate, 277 responded to the survey, giving an overall response rate of 19%. Representation from municipality organizations (*n* = 245; 88%) was predominant, followed by regional (*n* = 18; 7%) and private organizations (*n* = 14; 5%). We received responses from various locations throughout Sweden, suggesting a wide geographical spread. All 277 responses were complete with no missing data.

The majority of respondents were women (82.3%) with a college or university degree (Table 1). They came from various professions, although most were social workers or counselors with over 10 years of work experience. Most of the managers who had another core or primary profession (named “others,” 51.6%) were behaviorists, social pedagogues, human resources specialists, economists, political scientists, speech therapists, or teachers. However, the time worked at the current workplace was diverse, ranging from 1 year to more than 20 years. The number of clients served ranged from 0 to 14 000, and the number of employees managed varied from 0 to 1 500. Most managers held the position of “head of unit.” Additionally, more than half the managers were managers of both personal assistance and residential care services. A total of 11 managers were managers of all three services (habilitation, personal assistance, and residential care). The primary service areas were suburban areas with > 40 000 inhabitants, closely followed by suburban areas with > 15 000 inhabitants.

### TCU-ORC domains

The study found high scores (> 30) on the overall TCU-ORC survey, indicating that the managers perceived a high level of ORC and favorable conditions for implementing interventions. This was particularly the case within the domains *Resources*, *Staff Attributes*, and *Organizational Climate*. A low score (< 30) on scales such as *Program Needs* and *Training Needs* in the *Motivation for Change* domain indicated that the managers perceived little need for program improvements and training. The results in Table 2 show that the total mean TCU-ORC score was 36.68, suggesting the managers had a generally positive perception of their organization’s readiness for change across the sample. In the *Resources* domain, the *Internet* scale had the highest mean score. The *Training Needs* scale in the *Motivation for Change* domain had the lowest mean score.

**Table 2:** TCU-ORC scores

Domains	Scales	Managers of managers Mean score (SD)	Managers overseeing staff Mean score (SD)
Motivation for Change		31.62 (5.4)	31.68 (6.4)
To what degree managers perceive the organization’s need for guidelines and training, and where the pressure to change comes from.	Program Needs	29.43 (7.3)	31.11 (8.9)
	Training Needs	31.25 (7.0)	30.90 (8.1)
	Pressures for Change	34.17 (7.2)	33.04 (7.8)
Resources		39.49 (4.0)	37.86 (4.4)
Focuses on assessing the adequacy of institutional resources that support organizational functioning and change implementation	Offices	39.80 (8.4)	37.49 (9.6)
	Staffing	35.71 (5.7)	34.68 (6.9)
	Training	36.57 (6.4)	32.03 (8.2)
	Equipment	41.95 (4.8)	40.99 (5.4)
	Internet	43.40 (4.1)	44.10 (3.8)
Staff Attributes		42.31 (3.7)	40.85 (4.0)
Highlights the assessment of the characteristics, skills, and attitudes of staff members that contribute to an organization’s readiness for change	Growth	39.51 (6.0)	36.79 (7.5)
	Efficacy	42.41 (4.3)	41.77 (5.1)
	Influence	43.54 (4.6)	42.92 (5.0)
	Adaptability	42.09 (4.5)	41.59 (4.9)
	Satisfaction	44.01 (5.5)	41.20 (6.6)
Organizational Climate		37.69 (2.9)	35.63 (4.3)
Assesses the overall work environment and cultural aspects of an organization that can influence its readiness for change.	Mission	37.92 (4.7)	35.00 (7.1)
	Cohesion	38.30 (6.1)	36.38 (6.3)
	Autonomy	39.67 (4.6)	37.38 (6.5)
	Communication	38.24 (5.7)	35.40 (7.8)
	Stress	30.49 (7.0)	33.01 (9.4)
	Change	40.73 (5.8)	39.13 (6.4)
	Leadership	38.48 (4.3)	33.10 (8.2)
Total		37.78 (2.1)	36.51 (2.5)

*TCU-ORC* The Texas Christian University Organizational Readiness for Change

Overall, managers of managers tended to rate their organization’s implementation readiness more highly than managers overseeing staff directly. The multiple-regressions follow-up analyses including univariate significant variables showed few significant associations between the full TCU-ORC, domains, and scales and the socio-demographic and organization characteristics. However, there were significant differences between variables such as organization type, managerial level, tenure, client/employee ratios, and the four TCU-ORC domains (*Motivation for Change*,* Resources*,* Staff Attributes*, and *Organizational Climate*). Below, we summarize significant differences within the four domains (Tables 3, 4, 5 and 6).

**Table 3:** Multiple regression standardized coefficients (β) on background questions for the *Motivation for change* domain and its scales

Dependent variables	Domain	Scales		
	Motivation for Change	Program Needs	Training Needs	Pressure for Change
Sex (F/M)	-0.151**			-0.133*
Tenure	-0.173**	-0.293***	-0.141*	
Personal assistance services	0.242***	0.233***		0.195***
Residential area (< 40.000/>40.000)				0.183**

*p < 0.05, **p < 0.01, and ***p < 0.001

**Table 4:** Multiple regression standardized coefficients (β) on background questions for the *Resources* domain and its scales

Dependent variables	Domain	Scales			
	Resources	Staffing	Training	Equipment	Internet
Managers of managers	0.138*		0.223***		
Private organization	0.149*	0.152*		0.133*	
Residential care				-0.145*	-0.141*

*p < 0.05, **p < 0.01, and ***p < 0.001

**Table 5:** Multiple regression standardized coefficients (β) on background questions for the *Staff attributes* domain and its scales

Dependent variables	Domain	Scales				
	Staff Attributes	Growth	Efficacy	Influence	Adaptability	Satisfaction
Managers of managers	0.145*	0.135*				0.173**
Age		0.165**			-0.161**	
Tenure			0.133*	0.157**		
Habilitation service				0.140*		

*p < 0.05, **p < 0.01, and ***p < 0.001

**Table 6:** Multiple regression standardized coefficients (β) on background questions for the *Organization climate* domain and its scales

Dependent variables	Domain	Scales					
	Organization Climate	Mission	Cohesion	Autonomy	Communication	Stress	Leadership
Managers of managers	0.201***	0.149*	0.200**	0.142*	0.158**		0.273***
Residential care	-0.158**	-0.155**	-0.169**	-0.177**	-0.129*	0.124*	
Tenure		0.194**					
Number of employees			-0.172**				

*p < 0.05, **p < 0.01, and ***p < 0.001

#### Motivation for change

Overall, the *Motivation for Change* domain was rated more highly among female managers in personal assistance services who had been at the same organization for a shorter tenure (Table 3). A similar pattern was observed for the scales within the domain, except that the *Pressure for Change* scale was rated more highly among managers in areas with > 40 000 inhabitants. Thus, managers with shorter tenure in personal assistance services tended to respond more highly within the *Program Needs* scale, especially regarding the need to improve communication among staff. Similarly, those with a shorter tenure reported a higher rating within the *Training Needs* scale regarding new laws or regulations. Finally, the *Pressure for Change* scale was rated more highly among managers for personal assistant services. This was the only scale in this domain that showed significant gender and city size differences, with females and those in larger cities having higher ratings.

#### Resources

Managers of managers overseeing large numbers of employees compared with managers directly overseeing staff, as well as the managers working in private organizations compared with those working in municipal and region organizations, perceived their organization to have a higher degree of adequate resources (Table 4). Regarding the specific scales in the *Resources* domain, there were no significant associations related to *Offices*. However, significant associations were observed among the scales *Staffing*, *Training Resources*,* Equipment*,* Availability*, and *Internet* access. That is, managers in private organizations rated their *Staffing* and *Equipment* scales more highly than managers in municipal and region organizations. The highest scores were found for the *Staffing* scale, which measures whether the staff have the skills they need to do their jobs, and for the *Equipment* scale, which indicates whether computers are available. Residential care facilities had lower ratings within the *Equipment* and *Internet* scales compared with habilitation and personal assistant services.

#### Staff attributes

Managers of managers were the only predictor for the *Staff Attributes* domain (Table 5), particularly for the *Growth* and *Satisfaction* scales. The highest scores were within the *Growth* scale, which indicates whether the workplace encourages and supports professional growth, and the *Satisfaction* score, which indicates whether managers feel appreciated for the job they do and like the people they work with. Moreover, older managers gave higher ratings for their organization on the *Growth* scale and lower ratings on the *Adaptability* scale. Managers with more years of professional experience rated the *Efficacy* and *Adaptability* scales higher than managers overseeing employees directly. The highest rating for the *Efficacy* scale came from managers with the necessary skills to conduct their duties effectively, and the highest score for the *Adaptability* scale came from managers who could adapt quickly when needed to shift focus. Managers of habilitation services gave the highest rating for the *Influence* scale, with the maximum score coming from managers that frequently shared their knowledge or ideas with other staff.

#### Organizational climate

Managers of managers rated *Organizational Climate* more highly than managers directly overseeing employees. Managers from habilitation services gave the highest *Organizational Climate* ratings, followed by those from personal assistance services, while managers from residential care services gave their organizations the lowest rating (Table 6). For the *Mission* scale, managers of managers rated their organization’s clarity of goals and mission more highly than managers directly overseeing employees. The lowest score for clarity of goals and mission was given by managers from residential care services. The highest score within the *Mission* scale was given by managers who felt that their duties were clear and related to the overall goals. Managers of managers with fewer employees rated the *Cohesion* scale higher, compared with managers directly overseeing a larger staff, particularily within the residential care services. Managers who rated their organization highly on the *Cohesion* scale largely agreed that their staff members were always quick to help each other when needed. Within residential care, managers of managers also rated the *Autonomy* and *Communication* scales more highly than managers directly overseeing staff. The highest scores on the *Autonomy* scale were given by managers who felt that their managers fully trusted their professional judgment; the highest scores on the *Communication* scale came from managers who believed their staff could always ask questions and express concerns. Residential care managers directly overseeing staff were the only managers that gave a high rating for workload and stress. The only predictor for the *Leadership* scale within the *Organizational Climate* domain was the respondents being managers of managers, suggesting that these managers felt their management was good and their decisions were well planned. The highest score within the *Leadership* scale came from managers who considered that their staff participated in making long-range plans for the workplace.

### The results of responses to the open-ended questions

A total of 179 respondents (65%) answered the non-mandatory open-ended questions about enabling and barrier factors for implementation (Table 7). The analysis of enabling and barrier factors highlights critical organizational factors that influence implementation. Below is a summary of the key findings, categorized into enabling and barrier factors, and example of quotes.

**Table 7:** Categories and subcategories of the open-ended survey questions

Categories	Sub-categories	Quantified data within categories *n* (%)
Categories and sub-categories of factors enabling implementation		
Organizational prerequisites	Workplace culture	7 (3.6)
	Manageable staff groups	5 (2.6)
	Trust-based leadership	32 (16.6)
	Solution-focused participation	32 (16.6)
	Support functions	15 (7.8)
Competence and commitment	Knowledge and competence	12 (6.2)
	Training efforts	7 (3.6)
	Motivation and engagement	52 (27)
Resources and conditions	Financial resources	4 (2.0)
	Communication and collaboration	17 (8.8)
	Time	9 (4.6)
Categories and sub-categories of barrier factors for implementation		
Organizational challenges	Comprehensive decision paths	7 (3.0)
	Complex processes and lack of support	41 (17.8)
	Collaboration problems	6 (2.6)
Competence and change	Knowledge and competence	18 (9.3)
	Resistance and fear	35 (15.2)
	Workload	14 (6.0)
	Lack of time	33 (14.3)
	Financial and personnel resources	48 (20.8)
Communication and trust	Lack of communication	10 (4.3)
	Lack of trust	18 (9.3)

Enabling factors were cited 192 times and barrier factors 230 times within the categories

Several enabling factors were identified for the implementation of interventions. From a managerial perspective, enabling a sense of pride in the workplace and fostering a positive work environment are essential factors for a successful implementation. A sense of pride increases job satisfaction, and a positive work environment is characterized by good relationships between colleagues and empowering leadership. Managers showing confidence in their staff and being supportive enhance implementation success. To achieve this, it is important to have reasonable staff-to-work ratio to allow time for meetings, preparations, and discussions about implementation to foster a *trust-based leadership.*

Information, time, time to listen, positive spirit, to show confidence in the staff that they can do this. That I, as a manager, show that I am involved and supportive. (Municipal respondent)

Furthermore, managers need to offer relevant training, guidance, and opportunities for collegial learning and ensure adequate financial resources for implementation. Managers also emphasized the need to involve staff in decision-making processes and in *solution-focused participation.*

Participating employees. Good cooperation between manager and employees. Management and employees. Different forums for employees to work on the development of the operation. Time and resources. Good working environment. (Municipal respondent)

Furthermore, even if the managers’ highligted staff involvement they also stressed the need for employees to show *motivation and engagement* in work which appeared to enable the implementation of interventions.

Staff who take great personal responsibility for finding out relevant information, learning new things, and sharing with each other. Great interest in continuing education and further education—in the areas you are interested in yourself. A lot of time is set aside for meetings with colleagues, supervision, etc. (Regional respondent)

Conversely, barrier factors that can hinder implementation is *complex processes* in a bureaucratic apparatus (municipal administration).

The organization’s shortcomings, such as the hierarchy, bottlenecks, unclear goals, long decision-making paths, lack of knowledge “…” (Municipal respondent).

Management systems that do not talk to each other so there is a lot of duplication. Poor implementation of systems in the operations that cause concern. Poorly updated devices (Municipal respondent).

Furthermore, difficulties in creating collaboration across organizational boundaries, high workload and staff turnover, lack of communication and anchoring of decisions for implementation, and managers who lead remotely or implement changes too quickly without bringing employees along resulting in *resistance and fear* for implementation.

Unless those who are to perform and be involved have time to be shown by the manager in peace and quiet at a training or meeting. It never goes well when the responsibility is placed on staff to show each other ʺin everyday life.ʺ Everyone must have time to be shown around and have things explained so everyone is on board. Things that are thrown into the operation never turn out well, and it arouses resistance. (Municipal respondent)

Finally, a lack of financial and personnel resources was highlighted as a significant barrier.

Economy. In the assistance services, it is becoming more and more difficult to maintain the high quality desired at all levels, (…) There will soon be no opportunities for development in the area. (Private respondent)

### Combining TCU-ORC and open-ended question data

To complement the TCU-ORC data, the two open-ended questions highlighted enabling and barrier factors for organizations’ readiness to implement interventions. The enabling categories identified by managers in their responses to the open-ended questions—specifically, *trust-based leadership*, *staff involvement*, and *motivation and engagement—*support the TCU-ORC results. For example, the TCU-ORC measures *Organizational Climate*, which includes communication, autonomy, and openness to change—elements that may be crucial for fostering trust-based leadership [39]. Additionally, the TCU-ORC’s focus on *Staff Attributes*, including the scales *Growth*, *Efficacy*, and *Influence*, relates to an enabling factor identified in the responses to the open-ended questions: namely, involving staff in the implementation process. Furthermore, the enabling factors of motivating and engaging staff can be related to the *Motivation for Change* domain, which includes the organization’s overall need for guidelines and training as factors that may impact staff’s motivation for change. Similarly, the barriers identified in the open-ended question responses, including *complex processes*, *lack of support*, *resistance*, and *time and resource constraints*, correspond to the findings from the TCU-ORC. In the same way, The *Organizational Climate* domain evaluates an organization’s clarity of mission, where a lack of clarity could relate to barrier factor of *complex processes*. *Lack of support*, another barrier to implementation, could relate to the *Resources* domain, which includes skills, knowledge, and education, as weaknesses in these areas could indicate a lack of support. *Resistance* to change can be specifically related to the degree to which staff are open for change within the *Organizational Climate* domain. Lastly, identified barriers such as *resource constraints* are also reflected within the *Resources* domain under *Offices*, *Equipment*, and *Internet* access. In summary, the qualitative data from the open-ended questions complemented and clarified the quantitative results obtained from the TCU-ORC survey.

## Discussion

To the best of our knowledge, this is the first national study examining managers’ perceived ORC regarding the implementation of interventions within disability healthcare. The findings from the TCU-ORC data and the open-ended questions reveal a complex interplay of enabling and barrier factors for implementation readiness. Key enabling factors included trust-based leadership, staff involvement, motivation and engagement, aligning with existing research that emphasizes the importance of leadership support and employee engagement in fostering a readiness for implementation, this observation was particularly evident in other hospital settings [17, 19]. Qualitative studies have also highlighted the role of leadership [47], and for example within primary care trust in management, and perceived need for change in shaping managers’ perceptions of organizational readiness [20, 28]. Conversely, barriers to implementation encompassed complex processes, lack of support, resistance, and lack of time and resources, challenges identified within other hospital settings [48].

The study also highlighted the influence of individual managers’ characteristics, such as *gender*, *age*, *tenure*, *and experience*, on perceptions of ORC.

For example, female managers, those with shorter tenures, and those living in larger cities reported feeling greater pressure for change and the need for additional guidance and training. These findings have several implications for ORC. Female managers may perceive a greater need for support when implementing new interventions [49], highlighting the importance of providing tailored resources to enhance their readiness for change. These findings may be related to broader societal trends, such as the “glass cliff” phenomenon, where women are more likely to be appointed to leadership positions during challenging times [50]. This can be due to a lack of support and mentorship [51], work-life balance challenges [52], the impact of organizational culture on women’s experiences in leadership roles, and the fact that women often face higher performance standards and more negative evaluations than their male counterparts [53]. However, it is crucial to approach this interpretation cautiously, as the relationship between gender and leadership challenges is complex and influenced by many factors. Additionally, the difference in sample sizes between female and male respondents is an important factor to considered when interpreting and generalizing the results. The added complexity of being a manager in a larger urban area could contribute to this perceived pressure, as urban healthcare systems often face unique challenges due to population density, diversity, and resource demands [54].

Furthermore, managers with many employees and working in a private organization perceived greater resource adequacy and staff skills compared with managers with many employees in municipal and regional settings, suggesting that funding structure and resources allocation mechanisms significantly impact ORC. These findings is consistent with literature showing that resources availability is a key predictor for successful implementation [55]. Private organizations might have more flexibility in resource allocation, while public-sector organizations may face more budgetary constraints [56]. Additionally, private organizations compete in the market for resources and therefore may have greater economic incentives to reduce costs and operate efficiently and effectively. This market pressure may influence managers’ incentives to maximize organizational performance and effectiveness, potentially allowing more flexibility in decision-making in the private sector compared with the public sector [57].

The study also revealed the importance of managerial level and specific role challenges. Managers of managers reported greater professional growth and job satisfaction compared to managers directly overseeing staff, indicating that the increased responsibilities and broader scope of such roles can influence ORC. The managerial level is crucial for ORC as managers serve as key drivers, communicators, and supporters of change initiatives [19], significantly influencing employee attitudes and behaviors [58]. Job satisfaction plays a vital role in changing readiness, with satisfied employees being more likely to support and less likely to resist change, perceiving its benefits more positively. These findings align with other research studies on job satisfaction and professional growth in general [59, 60].

Age and experience were found to play a significant role in shaping managerial perspectives for ORC. Understanding managers’ age and experience is essential for assessing organizational readiness for change, as these factors significantly influence the change process. Older managers often exhibit greater readiness for change in terms of knowledge [61]. To support this, disability organizations can develop management strategies that leverage the strengths of different ages and experience profiles while addressing potential resistance through targeted interventions and intergenerational collaboration. Older managers value professional growth more than a willingness to try new ideas. More experienced managers felt that they had necessary skills and could adapt quickly. These findings suggest that age and experience differently influence managerial attitudes and skills [62]. That is, experience builds confidence in one’s skills and adaptability, while age shifts one’s focus to personal growth rather than innovation.

The study also highlighted specific challenges for residential care managers. Compared with managers of managers, the managers directly overseeing staff noted increased workload and stress, while perceiving less staff collaboration, communication, which could affect the readiness for implementation. This difference could be related to the unique challenges of managing care services, particularly considering the COVID-19 pandemic. The nature of residential care work may present specific challenges in terms of workload and stress management, in line with other studies examining burnout in residential aged care managers [63]. Increased workload and stress for residential care managers directly overseeing staff can significantly impact their ability for readiness, as they may struggle to provide the necessary support and guidance to staff during change. The perception of less staff collaboration, communication, and involvement in implementation indicates potential barriers for readiness [64]. The pandemic may have added new stressors and complexities to an already demanding work environment, potentially making staff more resistant to changes.

The enabling and barrier factors, together with the individual managers’ characteristics suggests a strong framework for understanding the complex interplay of factors for readiness. However, it is crucial to consider contextual factors [65] when examining enablers and barriers, as they can significantly impact ORC regarding the implementation of interventions in disability healthcare. This underscores the need for tailored approaches that address specific challenges while leveraging sector strengths. Future research should focus on comparative analyses to identify best practices for enhancing implementation readiness.

### Determinants within Weiner’s theory of organizational readiness for change

Weiner’s theory posits that organizational readiness is determined by the organization members’ shared belief in their collective ability to implement change, the value they assign to the change, and their belief that resources exist to support the change [16]. This study’s findings relate to Weiner’s theory, highlighting several key aspects that are supported by the results. Although Weiner focuses primarily on the organizational level, the findings from the present study highlight how individual factors such as gender, tenure, and age influence perceptions of readiness. This suggests that readiness may be better conceptualized as a multilevel construct involving interactions between both individual and organizational components.

The identified enablers and barriers in this study imply that readiness is not static but is a dynamic process that can shift over time as contextual factors change. Weiner’s theory could be expanded to incorporate this temporal dimension. Furthermore, the importance of trust-based leadership in enabling readiness goes beyond Weiner’s focus on organizational structures, suggesting that leadership style and behaviors may play a more central role in shaping readiness than originally theorized. The emphasis this study’s findings place on motivation and engagement as key enablers suggests that the psychological/motivational components of readiness may deserve greater attention in Weiner’s theory.

While Weiner acknowledges the importance of resource availability, our findings on lack of time and resource constraints as major barriers suggest that resource availability may be a more critical factor than portrayed in Weiner’s original theory. The identification of resistance as a key barrier suggests that it may be valuable to incorporate resistance more explicitly into the conceptualization of readiness. This addition would acknowledge the significant role resistance may play in hindering change efforts. The influence of contextual factors found in this study implies that readiness may be more context-dependent than Weiner’s general model suggests, since different interventions or organizational contexts may require unique considerations when assessing readiness.

### Limitations and strengths

The study’s response rate of 19% is relatively low, which raises concerns about potential non-response bias and the representativeness of the sample. The TCU-ORC is a long survey, consisting of 115 items. Its extensive length may have deterred participation and led to abandonment by respondents who found it too time-consuming to complete. Poorly timed survey distribution may also have reduced participation: The survey was sent out during a holiday period, which probably decreased the likelihood of potential respondents engaging with it. Asking managers to provide the email addresses of other managers may have created a risk of selection bias, as the managers could have chosen to provide contact information only for colleagues they have a good relationship with or who they believe will give positive responses—potentially causing the study to miss out on valuable insights from managers with different perspectives. Nevertheless, we wanted to reach out broadly to the organizations to avoid missing any potential respondents. The opinions expressed by the managers in the qualitative (second) phase of the study may have been influenced by the awareness of this topic when they carried out the quantitative (first) phase of the study [66]. A further limitation is that few responses were received from regional organizations. However, this can be explained by the fact that only 21 regions provide habilitation services in Sweden.

Despite these limitations, several factors suggest that this study still provides valuable insights. The good geographic distribution of responses across Sweden indicates that the sample may still capture a diverse range of perspectives. The lack of missing data suggests that the participants who did respond completed the survey fully, enhancing the quality of the collected data. The combination of quantitative and qualitative data provides a more comprehensive understanding of the subject matter [67]. Nevertheless, it is important to interpret the findings cautiously because nonresponse bias cannot be fully ruled out, and comparing the results of this study with those from similar studies is prudent in order to assess the generalizability of the findings.

### Conclusion

This study provides valuable insights into the factors influencing ORC regarding the implementation of interventions in disability services. Our findings revealed that managers’ perceptions of readiness are shaped by a complex interplay of personal, organizational, and contextual factors. The study also identified critical enabling factors such as trust-based leadership, staff involvement, and adequate resources, as well as barriers including complex processes and resistance to change. By employing a mixed-methods approach, this study provided a comprehensive and nuanced understanding of ORC regarding the implementation of interventions within disability healthcare, ultimately contributing to more effective implementation strategies and improved care for individuals with disabilities.

## Supplementary Information

Additional file 1. STROBE checklist.

Additional file 2. GRAMMS checklist.

## Data Availability

The datasets used and/or analysed during the current study are available from the corresponding author upon reasonable request.

## Abbreviations

GRAMMS: Good Reporting of a Mixed Methods Study
ORC: Organizational readiness for change
STROBE: Strengthening the Reporting of Observational Studies in Epidemiology
TCU-ORC: The Texas Christian University Organizational Readiness for Change
