# Supplementary material for: Managers’ perceptions of organizational readiness for change within disability healthcare: a Swedish national study with an embedded mixed-methods approach
Source: BMC Health Serv Res. 2025 May 6;25:648. doi: 10.1186/s12913-025-12808-4 (PMC12054221; doi:10.1186/s12913-025-12808-4)
Supplement: Supplementary file 1 — Additional file 1. STROBE checklist. [file 12913_2025_12808_MOESM1_ESM.pdf]

STROBE Statement—Checklist of items that should be included in reports of ***cross-sectional studies***

	Item No	Recommendation	Page No
**Title and abstract**	1	(*a*) Indicate the study's design with a commonly used term in the title or the abstract	Row 1-3
		(*b*) Provide in the abstract an informative and balanced summary of what was done and what was found	Row 32-54
**Introduction**			
Background/rationale	2	Explain the scientific background and rationale for the investigation being reported	Row 68-79<br>Row 111-114
Objectives	3	State specific objectives, including any prespecified hypotheses	Row 114-116
**Methods**			
Study design	4	Present key elements of study design early in the paper	Row 119-122
Setting	5	Describe the setting, locations, and relevant dates, including periods of recruitment, exposure, follow-up, and data collection	Row 126-130<br>Row 138<br>Row 141-147
Participants	6	(*a*) Give the eligibility criteria, and the sources and methods of selection of participants	Row 132-136
Variables	7	Clearly define all outcomes, exposures, predictors, potential confounders, and effect modifiers. Give diagnostic criteria, if applicable	Row 174-195
Data sources/ measurement	8*	For each variable of interest, give sources of data and details of methods of assessment (measurement). Describe comparability of assessment methods if there is more than one group	Row 161-172<br>Row 176-190
Bias	9	Describe any efforts to address potential sources of bias	Row 476-479<br>Row 517-519<br>Row 520-522
Study size	10	Explain how the study size was arrived at	Row 138-141
Quantitative variables	11	Explain how quantitative variables were handled in the analyses. If applicable, describe which groupings were chosen and why	Row 177-181
Statistical methods	12	(*a*) Describe all statistical methods, including those used to control for confounding	Row 204-211
		(*b*) Describe any methods used to examine subgroups and interactions	N/A
		(*c*) Explain how missing data were addressed	N/A
		(*d*) If applicable, describe analytical methods taking account of sampling strategy	N/A
		(*e*) Describe any sensitivity analyses	N/A
**Results**			
Participants	13*	(a) Report numbers of individuals at each stage of study—eg numbers potentially eligible, examined for eligibility, confirmed eligible, included in the study, completing follow-up, and analysed	Row 228-233<br>Row 151-152

		(b) Give reasons for non-participation at each stage	Row 228-229<br>Row 151-152
		(c) Consider use of a flow diagram	Row 151-152
Descriptive data	14*	(a) Give characteristics of study participants (eg demographic, clinical, social) and information on exposures and potential confounders	Row 233-244<br>Row 245-246
		(b) Indicate number of participants with missing data for each variable of interest	Row 232
Outcome data	15*	Report numbers of outcome events or summary measures	Row 248-257
Main results	16	(*a*) Give unadjusted estimates and, if applicable, confounder-adjusted estimates and their precision (eg, 95% confidence interval). Make clear which confounders were adjusted for and why they were included	Row 260-267
		(*b*) Report category boundaries when continuous variables were categorized	N/A
		(*c*) If relevant, consider translating estimates of relative risk into absolute risk for a meaningful time period	N/A
Other analyses	17	Report other analyses done—eg analyses of subgroups and interactions, and sensitivity analyses	Row 341-346
**Discussion**			
Key results	18	Summarise key results with reference to study objectives	Row 382-389
Limitations	19	Discuss limitations of the study, taking into account sources of potential bias or imprecision. Discuss both direction and magnitude of any potential bias	Row 501-515
Interpretation	20	Give a cautious overall interpretation of results considering objectives, limitations, multiplicity of analyses, results from similar studies, and other relevant evidence	Row 516-521
Generalisability	21	Discuss the generalisability (external validity) of the study results	Row 521-523
**Other information**			
Funding	22	Give the source of funding and the role of the funders for the present study and, if applicable, for the original study on which the present article is based	Row 550-551

*Give information separately for exposed and unexposed groups.

Note: An Explanation and Elaboration article discusses each checklist item and gives methodological background and published examples of transparent reporting. The STROBE checklist is best used in conjunction with this article (freely available on the Web sites of PLoS Medicine at http://www.plosmedicine.org/, Annals of Internal Medicine at http://www.annals.org/, and Epidemiology at http://www.epidem.com/). Information on the STROBE Initiative is available at www.strobe-statement.org.
