# Supplementary material for: Managers’ perceptions of organizational readiness for change within disability healthcare: a Swedish national study with an embedded mixed-methods approach
Source: BMC Health Serv Res. 2025 May 6;25:648. doi: 10.1186/s12913-025-12808-4 (PMC12054221; doi:10.1186/s12913-025-12808-4)
Supplement: Supplementary file 2 — Additional file 2. GRAMMS checklist. [file 12913_2025_12808_MOESM2_ESM.pdf]

Additional file 2 GRAMMS checklist* pdf

| Guideline | Section: page |
|---|---|
| Describe the justification for using a mixed methods approach to the research question | **Abstract**<br>**Background**<br>Section:<br>**Conclusion**<br>Lines: 51-53, 108-111 |
| Describe the design in terms of the purpose, priority and sequence of methods | **Methods**<br>Section:<br>**Study design**<br>Lines: 119-122 |
| Describe each method in terms of sampling, data collection and analysis | **Methods**<br>Section:<br>**Data collection**<br>**Data analysis**<br>Lines: 138-201, 202-223 |
| Describe where integration has occurred, how it has occurred and who has participated in it | **Methods**<br>Section:<br>**Data integration**<br>Lines: 199-201 |
| Describe any limitation of one method associated with the present of the other method | **Discussion**<br>Section:<br>**Limitations and Strengths**<br>Lines: 511-513 |
| Describe any insights gained from mixing or integrating methods | **Discussion**<br>Section:<br>**Limitations and Strengths**<br>Lines: 518-521 |

*O'Cathain A, Murphy E, Nicholl J. The quality of mixed methods studies in health services research. J Health Serv Res Policy. 2008;13: 92-98.
